# Near-chromosome level genome assembly reveals ploidy diversity and plasticity in the intestinal protozoan parasite *Entamoeba histolytica*

**DOI:** 10.1186/s12864-020-07167-9

**Published:** 2020-11-23

**Authors:** Tetsuro Kawano-Sugaya, Shinji Izumiyama, Yasuaki Yanagawa, Yumiko Saito-Nakano, Koji Watanabe, Seiki Kobayashi, Kumiko Nakada-Tsukui, Tomoyoshi Nozaki

**Affiliations:** 1 Graduate School of Medicine, The University of Tokyo, Bunkyo, Tokyo, Japan; 2 Department of Parasitology, National Institute of Infectious Diseases, Shinjuku, Tokyo, Japan; 3 AIDS Clinical Center, National Center for Global Health and Medicine, Shinjuku, Tokyo, Japan; 4 Department of Infectious Diseases, Keio University School of Medicine, Shinjuku, Tokyo, Japan

**Keywords:** *Entamoeba*, Aneuploidy, Expression regulation, Hi-C, PacBio

## Abstract

**Background:**

Amoebozoa is a eukaryotic supergroup composed of unicellular and multicellular amoebic protozoa (e.g. *Acanthamoeba*, *Dictyostelium*, and *Entamoeba*). They are model organisms for studies in cellular and evolutionary biology and are of medical and veterinary importance. Despite their importance, Amoebozoan genome organization and genetic diversity remain poorly studied due to a lack of high-quality reference genomes. The slime mold *Dictyostelium discoideum* is the only Amoebozoan species whose genome is available at the chromosome-level.

**Results:**

Here, we provide a near-chromosome-level assembly of the *Entamoeba histolytica* genome, the second semi-completed Amoebozoan genome. The availability of this improved genome allowed us to discover inter-strain heterogeneity in ploidy at the near-chromosome or sub-chromosome level among 11 clinical isolates and the reference strain. Furthermore, we observed ploidy-independent regulation of gene expression, contrary to what is observed in other organisms, where RNA levels are affected by ploidy.

**Conclusions:**

Our findings offer new insights into *Entamoeba* chromosome organization, ploidy, transcriptional regulation, and inter-strain variation, which will help to further decipher observed spectrums of virulence, disease symptoms, and drug sensitivity of *E. histolytica* isolates.

**Supplementary Information:**

The online version contains supplementary material available at 10.1186/s12864-020-07167-9.

## Background

*Entamoeba histolytica* is an intestinal protozoan parasite that causes human amebiasis. It is a major causative agent of diarrheal diseases, which was ranked fifth in the 2015 list of diseases responsible for high disability-adjusted life years (DALYs) [1]. *E. histolytica* resides in the large intestine, which represents an anaerobic environment. Thus, it has evolved unique core metabolism, mitochondrial structure and function, and cellular activities, such as compartmentalized sulfate activation in the mitochondrion-related organelle (mitosome) [2] and internalization of live mammalian cells by trogocytosis [3].

Due to its medical importance and biological peculiarities, the first draft genome of the *E. histolytica* HM-1:IMSS reference strain was reported in 2005 [4]. The first reported assembly was 23.8 Mb long with 12.5-fold coverage and was segmented into 888 scaffolds. In the latest public database [5], the assembly is segmented into 1496 scaffolds. Unfortunately, the genome structure predicted by the first draft was highly fragmented, due to the repetitive nature of the genome (e.g. short interspersed nuclear elements (SINEs), long interspersed nuclear elements (LINEs), tRNA arrays containing short tandem repeats [6], segmental duplications, and polyploidy), which complicates chromosome-level genome characterization.

## Results

**Fig. 1:**
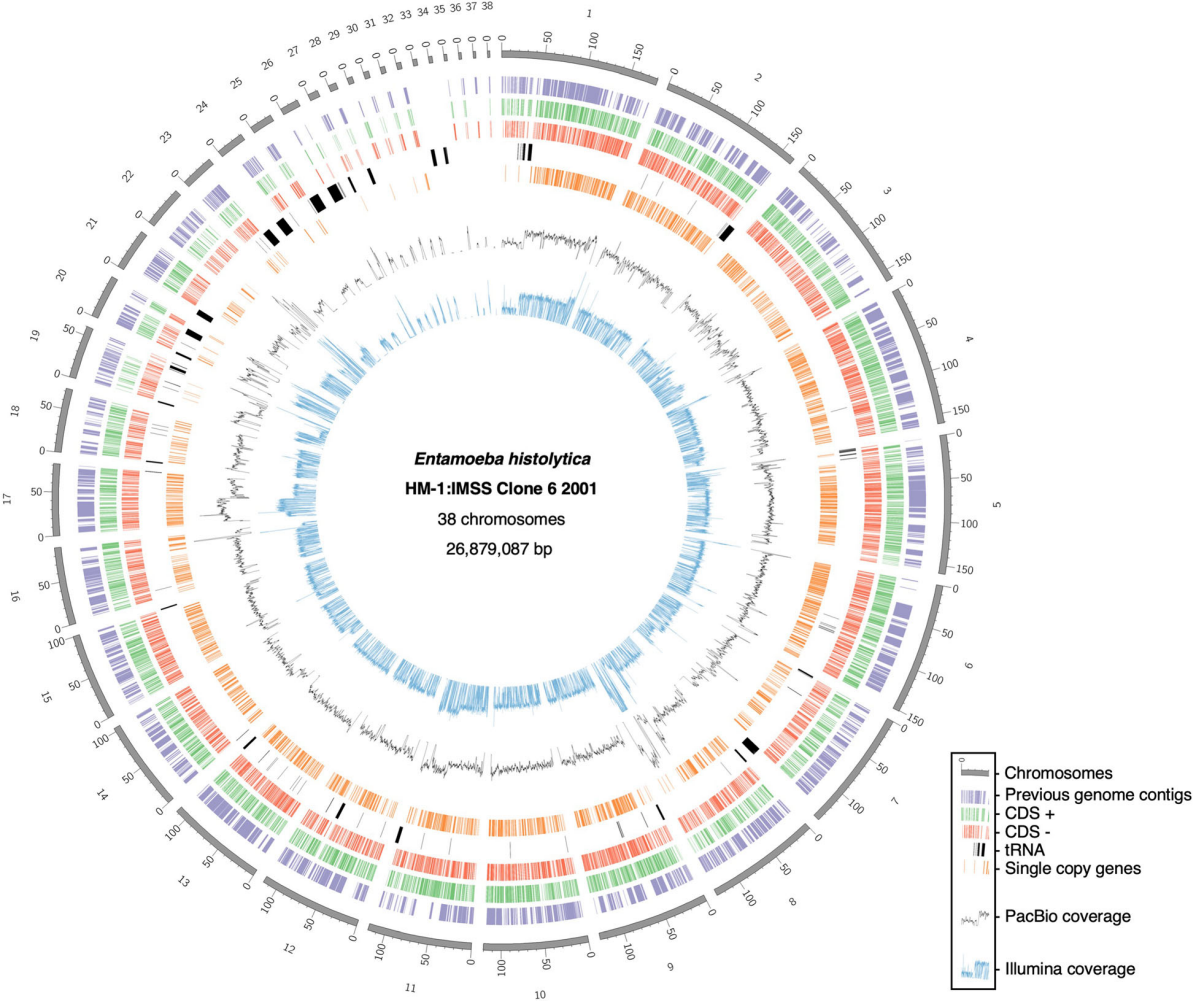
Overview of the genome of *E. histolytica* HM-1:IMSS Clone 6 2001. This circos plot shows overall structures with length, annotations, and coverage graphs of PacBio and Illumina reads of the genome of *E. histolytica* HM-1:IMSS Clone 6 2001. From outside to inside, newly assembled scaffolds and their length (gray blocks; with a scale of 1:10 kb), the previous genome contigs (purple bar codes), coding sequences + strand (green bar codes), coding sequences - strand (orange bar codes), tRNA genes (black bar codes), single copy genes (orange bar codes), PacBio read coverage (a black line), and Illumina read coverage (a blue line) are shown

We assembled the *E. histolytica* HM-1:IMSS Clone 6 2001 genome ab initio with near-chromosome level resolution utilizing PacBio and Hi-C [7]. The assembly workflow is shown in Extended Data 1 (Additional file 1). Briefly, we obtained reads with ~ 409-fold coverage by PacBio SMRT sequencing and ~ 963-fold by Hi-C. After construction of several assemblies using HGAP3 [8], HGAP4, and Flye [9], HGAP3 was the most redundant and lossless assembly, representing 35 Mb fragmented into 403 contigs that were scaffolded with Hi-C reads. The near-chromosome level scaffolds were arranged by JuiceBox [10], and gaps were filled using PBjelly [11]. The resultant genome is composed of 38 scaffolds with a length of 26,879,087 bp. The genome contains a total of 8734 protein-coding genes according to prediction by Companion [12] without RNA-seq data. The number of scaffolds is comparable to that suggested by a previous study, in which pulse-field gel electrophoresis was exploited (31–35 chromosomes of over 300 kb) [13]. An overview of all scaffolds is shown in Fig. 1. A total of 1421 of the 1496 contigs from the previous genome assembly were mapped to the newly assembled genome by BLAST [14] (top hits were indicated with purple barcodes in the outer circle in Fig. 1). Among the remaining 75 contigs, 73 were very short (1 ~ 3 kb) and two were contamination of the extrachromosomal plasmid [15].

**Fig. 2:**
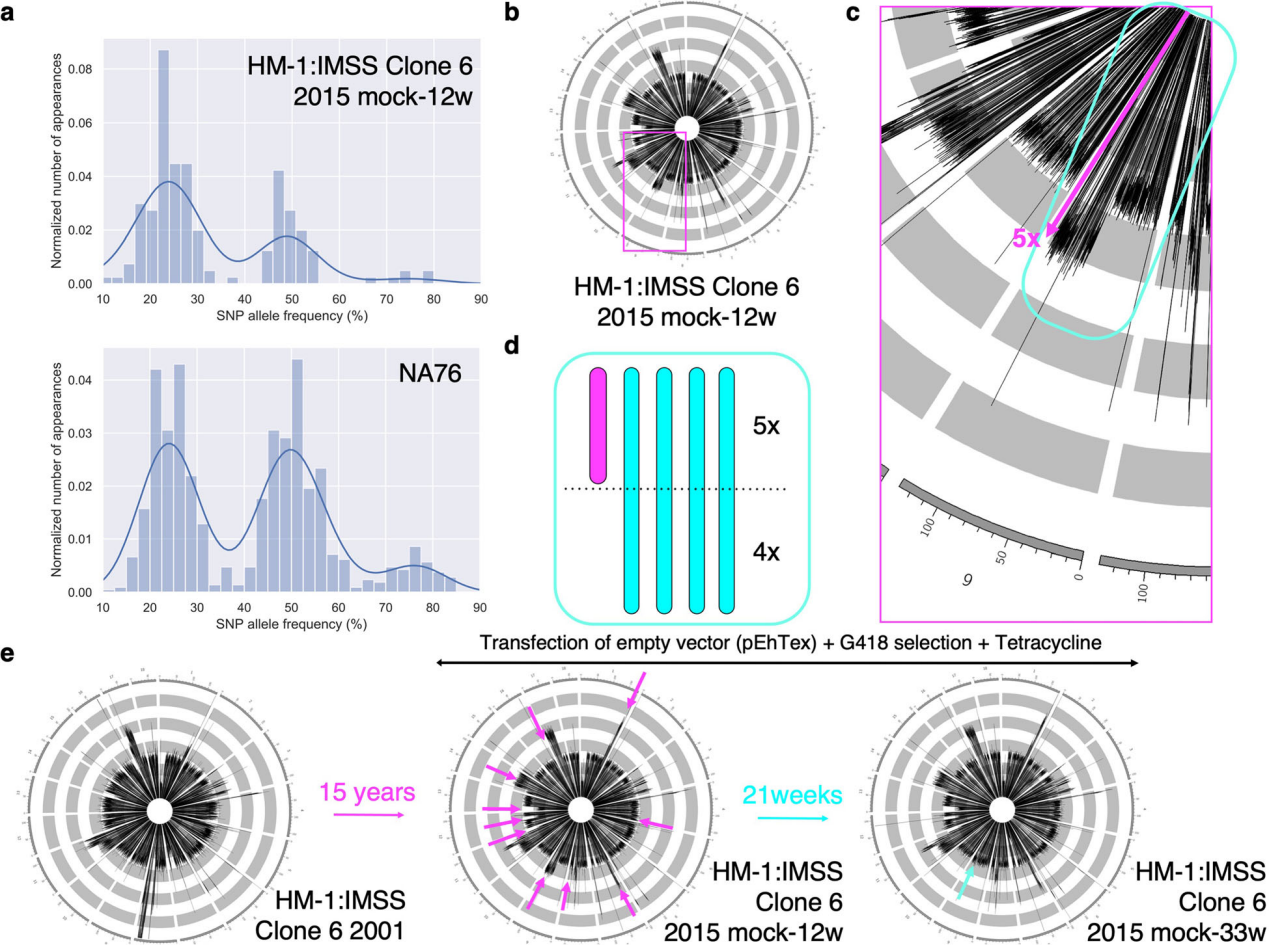
The ploidy patterns of *E. histolytica* HM-1:IMSS Clone 6 2015 mock-12w and NA76. **a,** Histograms of SNP allele frequencies (%) vs. normalized numbers of genes (proportions of the total genes) analyzed in HM-1:IMSS Clone 6 2015 mock-12w (upper panel) and NA76 (lower panel). They show peaks at 25, 50, and 75% of allele frequencies in both strains, suggesting that the genome is tetraploid. **b,** An example of the ploidy pattern in HM-1:IMSS Clone 6 2015 mock-12w, suggesting that the ploidy of *E. histolytica* is 4 at large, but fluctuates in a chromosomal and sub-chromosomal specific fashion. **c,** The magnified image of the rectangle in magenta in Fig. 2b. Scaffold 9 is highlighted with a cyan rectangle. Based on the read mapping, the first half of scaffold 9 is tetraploid, whereas the second half is pentaploid (indicated with a magenta arrow). **d,** A schematic model of the ploidy of scaffold 9. There are four identical chromosomes of full length and one partial (cropped) chromosome corresponding to the second half of scaffold 9. **e,** The comparison of ploidy patterns among three isogenic strains: HM-1:IMSS Clone 6 2001, 2015 mock-12w, and 2015 mock-33w. The ploidy patterns altered during 15 years of cultivation (magenta arrows), but the tetraploidy is kept at large. On the other hand, only slight difference was observed after 21 additional weeks of cultivation after transfection of a mock empty plasmid in the presence of G418 (an cyan arrow)

Using this newly assembled genome, we examined the chromosome organization and ploidy of *E. histolytica*. In order to better understand *E. histolytica* ploidy, we examined the allelic frequencies of SNPs across the whole genome. We collected and selected high probability SNPs from the HM-1:IMSS Clone 6 2001 genome using the Fixed Ploidy Variant Detection tool in CLC Genomics Workbench 8.5.1 (QIAGEN). The normalized SNP numbers were plotted against the allele frequencies, and a histogram shows three well-separated peaks at approximately 25, 50, and 75% (Fig. 2a, top panel). These data are consistent with the premise that the *E. histolytica* genome is tetraploid. We confirmed that other representative *E. histolytica* strains (NA22, NA53, and NA76) also show a similar distribution pattern of SNP allelic frequencies (Fig. 2a, Extended Data 2 (Additional file 1)), further supporting the claim [13].

We further investigated if ploidy varies at the chromosomal and/or sub-chromosomal levels. We mapped the Illumina reads of the HM-1:IMSS Clone 6 2001 assembly to the new *E. histolytica* genome assembled herein. We found that the coverage varies between scaffolds and between different regions on the same scaffold (Fig. 2b and c). The relative level of coverage indicates the number of homologous chromosomes (or chromosomal regions) that contain the gene. We normalized the whole coverage to be four, based on the assumption that the genome is tetraploid according to previous studies and as discussed above [13]. Accordingly, some scaffolds and some regions of scaffolds occasionally show a relative coverage of five, six, or seven. These results indicate that the genome is aneuploid; it is tetraploid in general, but is occasionally > 4-ploid at the chromosomal and sub-chromosomal levels and contains one or more additional copies of the shorter truncated chromosome, as shown in the example in Fig. 2d.

We investigated if the ploidy pattern is stable during in vitro cultivation. We compared the ploidy pattern between HM-1:IMSS Clone 6 2001 and HM-1:IMSS Clone 6 2015 mock-12w, the latter of which was derived from HM-1:IMSS Clone 6 2001, maintained in vitro in BI-S-33 medium for 15 years [16], transfected with an empty vector (pEhTex) [17, 18], and cultivated in vitro in the presence of 6 μg/mL G418 and 10 μg/mL tetracycline. The data show subtle but significant differences in the ploidy pattern between these two isogenic strains. We also compared the ploidy pattern between HM-1:IMSS Clone 6 2015 mock-12w and HM-1:IMSS Clone 6 2015 mock-33w, the latter of which was cultured for an additional 21 weeks in BI-S-33 medium containing G418 and tetracycline. Slight alterations to the ploidy pattern were found after the additional 21-week cultivation (Fig. 2e).

**Fig. 3:**
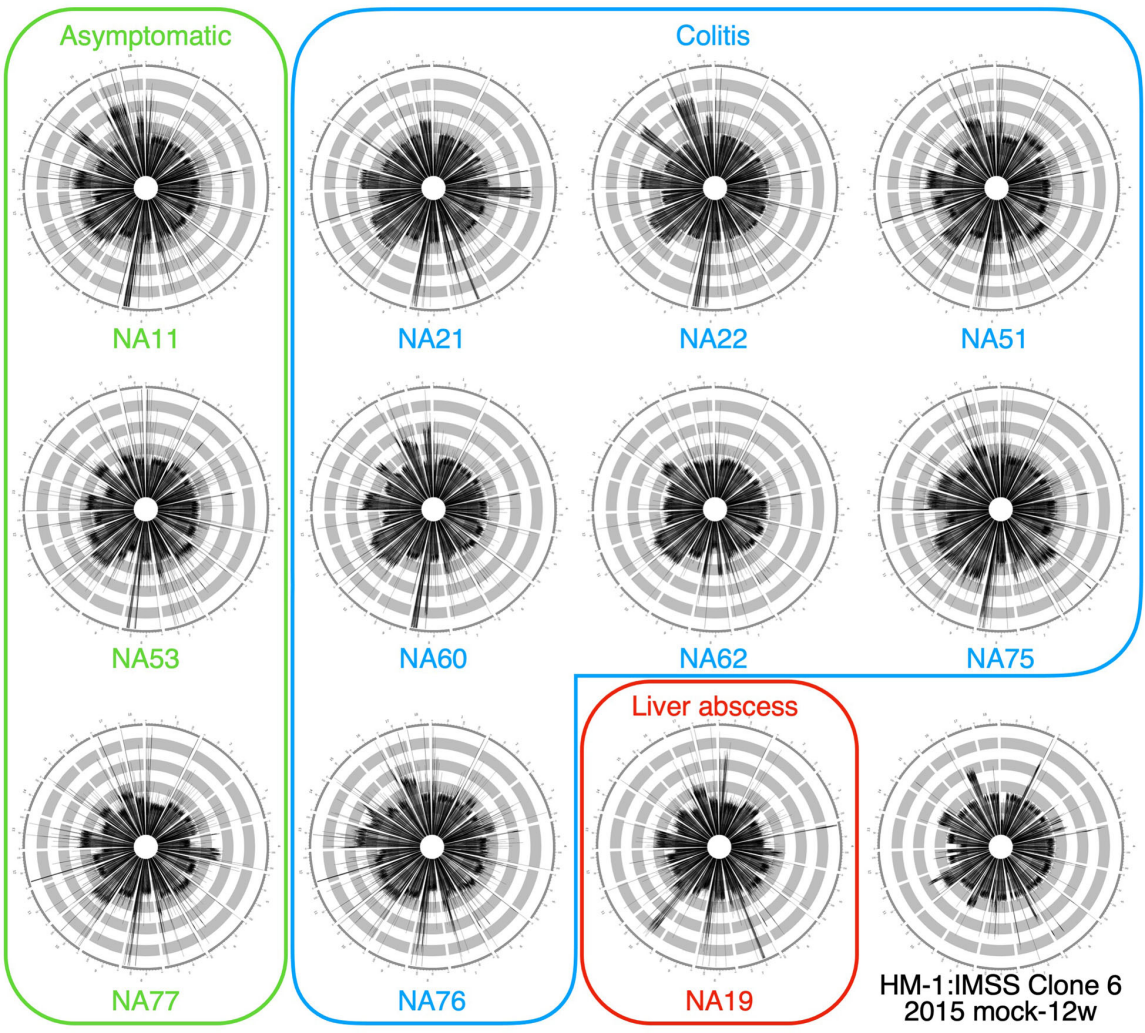
Ploidy patterns of 11 *E. histolytica* clinical isolates. The ploidy patterns of clinical isolates. The patterns of asymptomatic strains (NA11, NA53, and NA77), colitis strains (NA21, NA22, NA51, NA60, NA62, NA75, and NA76), and a liver abscess strain (NA19) are shown in green, blue and red rectangle, respectively. The pattern of HM-1:IMSS Clone 6 2015 mock-12w is also shown. All strains show different ploidy patterns, suggesting no direct correlation between the ploidy patterns and the clinical symptoms

We next examined the ploidy patterns of 11 *E. histolytica* clinical strains isolated from patients with different clinical manifestations [Extended Data 4 (Additional file 1)]. Surprisingly, all clinical strains show distinct ploidy patterns which seem to be symptom-independent (Fig. 3).

**Fig. 4:**
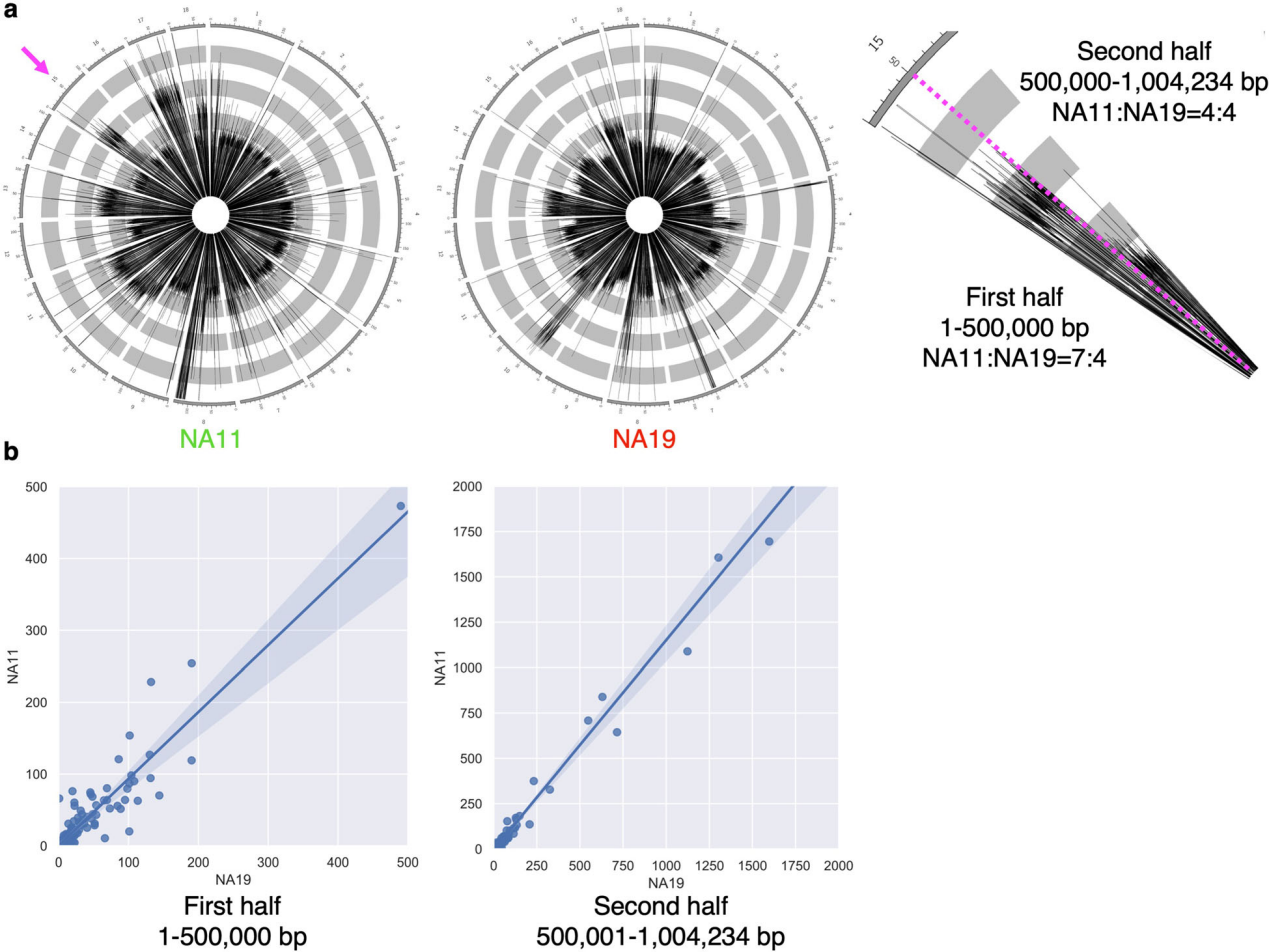
Lack of correlation between ploidy and RNA level. **a,** The ploidy patterns of scaffold 15 (indicated by an magenta arrow) of NA11 and NA19. In NA11, the first half of scaffold 15 is septaploid, whereas the second half is tetraploid (left). In NA19, the entire scaffold 15 is tetraploid (middle). The magnified image of the corresponding area of NA11 in the left panel is shown in the right panel. **b,** Plots showing a linear association of the relative abundance of RNA, shown in RPKM values, of the genes located in the first (left) and second (right) halves of scaffold 15 between NA11 and NA19

Finally, we examined if ploidy patterns are associated with differential gene expression. Specifically, we attempted to determine whether all or only a subset of the homologous chromosomes is transcriptionally active. To test this, we compared RNA-seq data with genome mapping data from the NA11 and NA19 representative clinical strains. We chose scaffold 15 because its ploidy pattern is unstable and is thus appropriate for comparison. Genome mapping data indicate that in the NA11 strain, the first half of scaffold 15 is septaploid (7x) while the second half is tetraploid. However, in the NA19 strain, scaffold 15 is homogeneously tetraploid (Fig. 4a). We compared the RPKM values of all genes encoded in the first and second halves of scaffold 15 between the NA11 and NA19 strains (Fig. 4b). Interestingly, the observed variations in ploidy do not correlate with mRNA levels (Fig. 4b).

## Discussion

Here, we have provided the improved reference genome of *E. histolytica* HM-1:IMSS Clone 6 2001 using PacBio and Hi-C. According to assembly statistics and BLAST barcoding analysis, our assembly appears to have significantly improved quality compared to the previously genome [4]. The new reference genome should help in the understanding of genome architecture, synteny between strains and species, SNPs discovery, and all downstream research.

In this study, we did not extract and analyze SNPs across strains. This decision was made based on our observation that almost all SNPs were located outside of open reading frames (ORFs) and no heterozygosities were found in ORFs, despite high gene density. The fact suggests the existence of strict selection pressures on the coding sequences in contrast to the plasticity of the intergenic regions. Nevertheless, our assembly enabled us to depict the diversity in the ploidy among *E. histolytica* strains. Ploidy varies widely between species, between cells, tissues, and organs of a given species, and also sometimes between individuals. The biological implications of ploidy are also diverse. It is well established that ploidy abnormalities can cause death or severe disorder in multicellular organisms. It is also known that changes to ploidy may act as a strong force driving evolution [19]. Amoebozoa is an interesting eukaryotic superfamily containing organisms with normal to extreme variations in ploidy: e.g. *Acanthamoeba castellanii* (< 25-ploid) [20], *Amoeba proteus* (500 ~ 1000-ploid) [21], and *Polychaos dubium* (1150 ~ 23,000-ploid) [21]. The ploidy of *E. histolytica* is variable because their chromatin does not condense in all stages of its life cycle. However, it was presumed, based on pulse-field gel electrophoresis and Southern hybridization using single copy gene probes, that *E. histolytica* had at most four sets of homologous chromosomes that may vary in length among strains [13].

Variances in ploidy are observed in cancer cells, which contained mutations in several key genes involved in cell cycle control and chromosome segregation [22]. It was also reported that *Leishmania*, another group of protozoa responsible for visceral and (muco)cutaneous leishmaniasis, possesses such chromosomal mosaicism which is likely caused by errors in chromosome replication [23]. It is worth noting that the *E. histolytica* genome lacks some key genes involved in cell cycle control and chromosome segregation (e.g. components of APC/C, cohesins, condensins, and kinetochore; Extended Data 3 (Additional file 1)). Of importance are the APC/C regulator Rae1 and Nup98, whose loss causes severe aneuploidy in mice [24]. Rae1 and Nup98 seem to be functionally lost from the *E. histolytica* genome according to BLAST e-values [Extended Data 3 (Additional file 1)].

Another important discovery of our study was that ploidy patterns are apparently unstable. Our data suggest that ploidy patterns at the near-chromosome level change during in vitro cultivation, while tetraploidy is maintained in general. Ploidy patterns may change upon adaptation to different environmental conditions such as hosts, organs, drugs, and intestinal microbiota. Alternatively, variations in ploidy patterns may simply reflect the genomic heterogeneity of the clinical strains. These data argue against the possibility that ploidy patterns are directly related to virulence in humans.

We observed no relationships between ploidy and expression levels in *E. histolytica*. This is in stark contrast to cancer cells and *Leishmania*, where DNA content and mRNA expression of some genes were correlated [23]. These data suggest that not all homologous chromosomes (or loci) may not be transcriptionally active in *E. histolytica*. Alternatively, one possible explanation for the lack of correlation between the ploidy number and the steady-state transcript levels is that while genes on homologous chromosomes are unbiasedly transcribed, there are some feedback mechanisms such as (1) RNA destabilization and degradation or (2) transcriptional repression via transcriptional gene silencing with antisense small RNA, as demonstrated in this organism [25]

## Conclusions

In summary, we significantly improved the currently available *E. histolytica* reference genome by combining sequencing data from three NGS platforms. We demonstrated that the ploidy of isogenic strains is dynamic upon in vitro cultivation, and that ploidy patterns also vary among clinical strains. We also uncovered a mechanism that uncouples ploidy and gene expression level and leads to variations in genome structure and ploidy patterns, which may underly variation in parasite virulence. These findings have implications in the virulence spectrum and drug sensitivities observed among clinical parasite strains that cause a range of disease manifestations.

## Methods

### Organisms, cultivation, and DNA extraction

Trophozoites of isogenic *E. histolytica* strains (HM-1:IMSS Clone 6 2001, 2015 mock-12w, and 2015 mock-33w) were cultured in BI-S-33 medium [15]. A total of 11 clinical strains were obtained from outpatients were diagnosed for amebiasis at the AIDS Clinical Center (ACC) of the National Center for Global Health and Medicine (NCGM) in 2014–2017. The details regarding the culture medium, date of isolation, clinical manifestations of the patients and additional descriptions, as well as the genome sequencing platforms used are shown in Extended Data 4 (Additional file 1).

### Illumina data generation

Genomic DNA was extracted from approximately 10 [7] cells of *E. histolytica* trophozoites using the QIAGEN Blood & Cell Culture DNA Kit with Genomic-tip 100/G (QIAGEN GmbH, Germany). Nuclei were isolated according to the manufacturer’s instructions (QIAGEN Genomic DNA Handbook). Purity and quantity of DNA samples were estimated using the Qubit dsDNA HS Assay Kit (Thermo-Fisher Scientific, Massachusetts, US).

### PacBio data generation

A total of 30.3 μg of genomic DNA was extracted from approximately 3.0 × 10 [8] cells from *E. histolytica* HM-1 Clone 6 2001 trophozoites using the QIAGEN Blood & Cell Culture DNA Kit with Genomic-tip 100/G (Qiagen GmbH, Germany). The integrity of high molecular weight DNA was confirmed by agarose-gel electrophoresis. DNA templates of an average size > 20 kb were produced using the BluePippin Size-Selection System Template Prep Kit and the SMRTbell Template Prep Kit 1.0. The DNA templates were sequenced on 9 SMRT cells of the PacBio RS II (Pacific Biosciences, California, US) by the Okinawa Institute of Advanced Sciences (Okinawa, Japan). The SMRTbell adapters were removed from raw reads and filtered for quality by the institute.

### Hi-C data generation

A total of 1.2 × 10 [8] log-phase trophozoites were collected by centrifugation at 500 x g for 5 min at 4 °C and washed 3 times with phosphate buffered saline (PBS). Cells were resuspended in 10 mL of PBS and mixed with 40 mL of buffer C1 (QIAGEN) in a 50 mL plastic conical tube. The tube was gently inverted 8 times and incubated on ice for 10 min. After the incubation, nuclei were collected by centrifugation at 1300 x g for 10 min at 4 °C, followed by resuspension in 10 mL of PBS. Isolated nuclei were crosslinked with 1% formaldehyde at room temperature (RT) for 15 min, with intermittent inversion of the tube. Excess formaldehyde was quenched by incubation with a final concentration of 1% (w/v) glycine for 15 min at RT by adding glycine powder. Fixed nuclei were washed with PBS, collected by centrifugation at 1500–2000 x g for 10 min at 4 °C, and stored frozen at − 30 °C until use. The frozen nuclei were analyzed by the commercial scaffolding service, Proximo Hi-C Genome Scaffolding, provided by Phase Genomics, Inc. (Seattle, Washington, US). Their draft scaffolds and raw reads were used for latter analyses.

### Assembly of the HM-1:IMSS clone 6 2001 reference genome

The workflow for genome assembly is shown in a flow diagram [Extended Data 1 (Additional file 1)]. Briefly, genomic DNA extracted from nuclei was sequenced by PacBio RSII, and the reads were assembled using three assemblers: HGAP3, HGAP4, and Flye. For downstream analyses, we used the assembly created by HGAP3 because it was thought to be the most redundant and lossless assembly. The assembly was scaffolded using Hi-C linked reads by the Proximo Genome Scaffolding service. After scaffolding, we filled in gaps in the assembly using PBjelly. The output was manually corrected using Juicer and Juicebox, and gap filling was performed again. Finally, 38 scaffolds and one circular plasmid were assembled. The genome was temporally annotated by Companion.

### SNP calling

Using the new assembly as a reference sequence, we ran the Fixed Ploidy Variant Detection tool in CLC Genomics Workbench 8.5.1. Ploidy was fixed to four according to our results and a previous study [8]. The program returns the list of heterozygous SNPs with index values of probabilities and reliabilities (QUAL). After filtering the results for QUAL = 200 (maximum), we manually verified the SNPs using readmapping. Then, the allele frequencies of those SNPs were drawn as bar-charts as shown in Fig. 2a and Extended data 2 (Additional file 1).

## Supplementary Information

**Additional file 1:** Extended Data 1-4.

## Data Availability

The genome assembly and sequence data for HM-1:IMSS Clone 6 2001 and other strains in this study were deposited at DNA Data Bank of Japan (DDBJ) under AP023109-AP023147, PRJDB8679, and PRJDB8495.

## Abbreviations

RNA: Ribonucleic acid
DALY: Disability-adjusted life years
SINE: Short interspersed nuclear elements
LINE: Long interspersed nuclear elements
SMRT: Single molecule real-time sequencing
BLAST: Basic local alignment search tool
SNP: Single nucleotide polymorphism
ORF: Open reading frames
APC/C: Anaphase promoting complex/cyclosome
RPKM: Reads per kilobase of exon per million mapped sequence reads
DNA: Deoxyribonucleic acid
NGS: Next-generation sequencing
ACC: AIDS Clinical Center
NCGM: National Center for Global Health and Medicine
PBS: Phosphate buffered saline
RT: Room temperature
DDBJ: DNA Data Bank of Japan
NIID: National Institute of Infectious Diseases
AMED: Japan Agency for Medical Research and Development
J-GRID: Japan Initiative for Global Research Network on Infectious Diseases
SATREPS: Science and Technology Research Partnership for Sustainable Development
JICA: Japan International Cooperation Agency
