# Supplementary figures and images for: Near-chromosome level genome assembly reveals ploidy diversity and plasticity in the intestinal protozoan parasite Entamoeba histolytica
Source: BMC Genomics. 2020 Nov 23;21:813. doi: 10.1186/s12864-020-07167-9 (PMC7681961; doi:10.1186/s12864-020-07167-9)

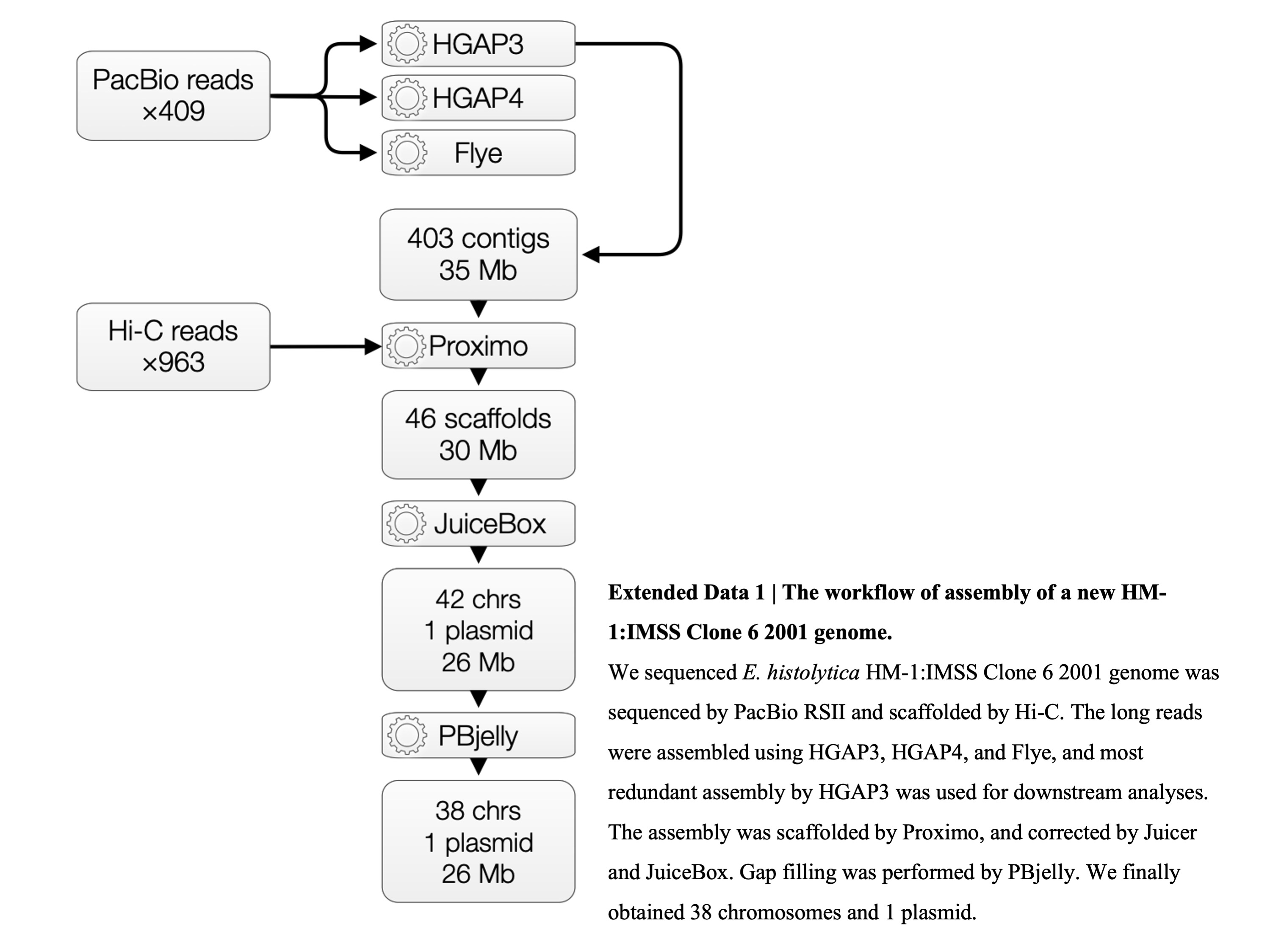

Supplement: Supplementary file 1 — Additional file 1: Extended Data 1-4. [file 12864_2020_7167_MOESM1_ESM.zip › 12864_2020_7167_MOESM1_ESM/extdata1.jpeg]

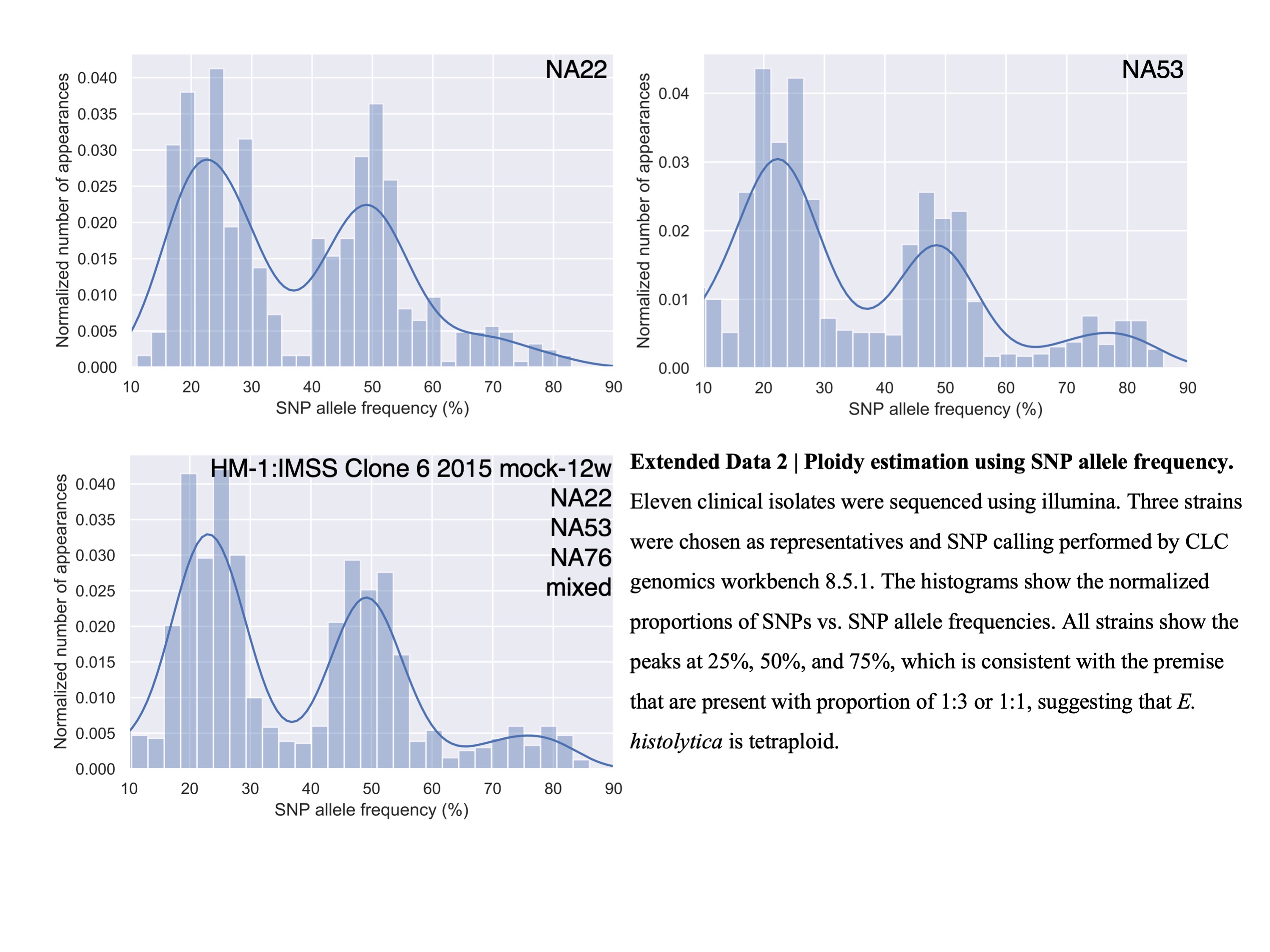

Supplement: Supplementary file 1 — Additional file 1: Extended Data 1-4. [file 12864_2020_7167_MOESM1_ESM.zip › 12864_2020_7167_MOESM1_ESM/extdata2.jpeg]

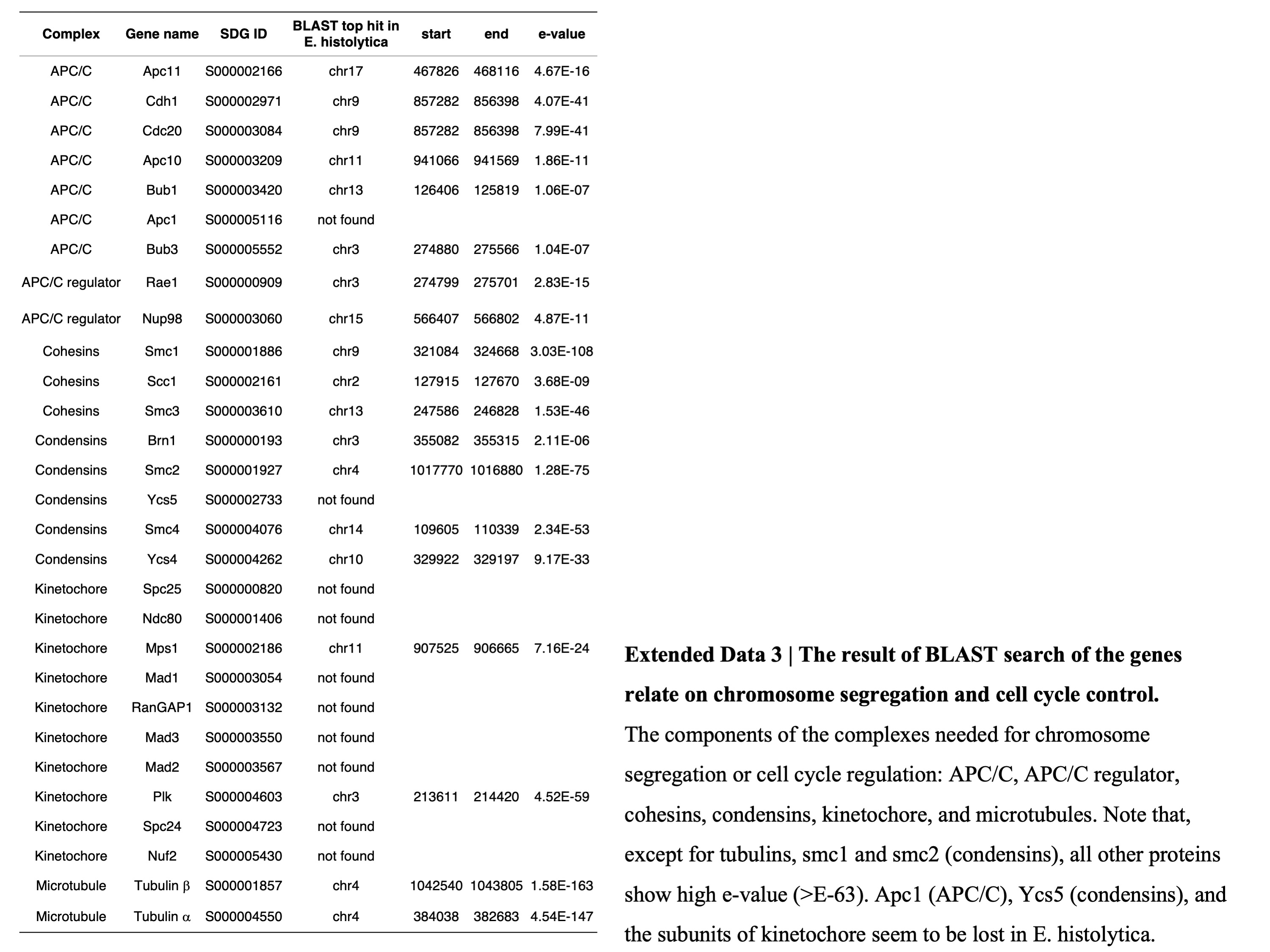

Supplement: Supplementary file 1 — Additional file 1: Extended Data 1-4. [file 12864_2020_7167_MOESM1_ESM.zip › 12864_2020_7167_MOESM1_ESM/extdata3.jpeg]

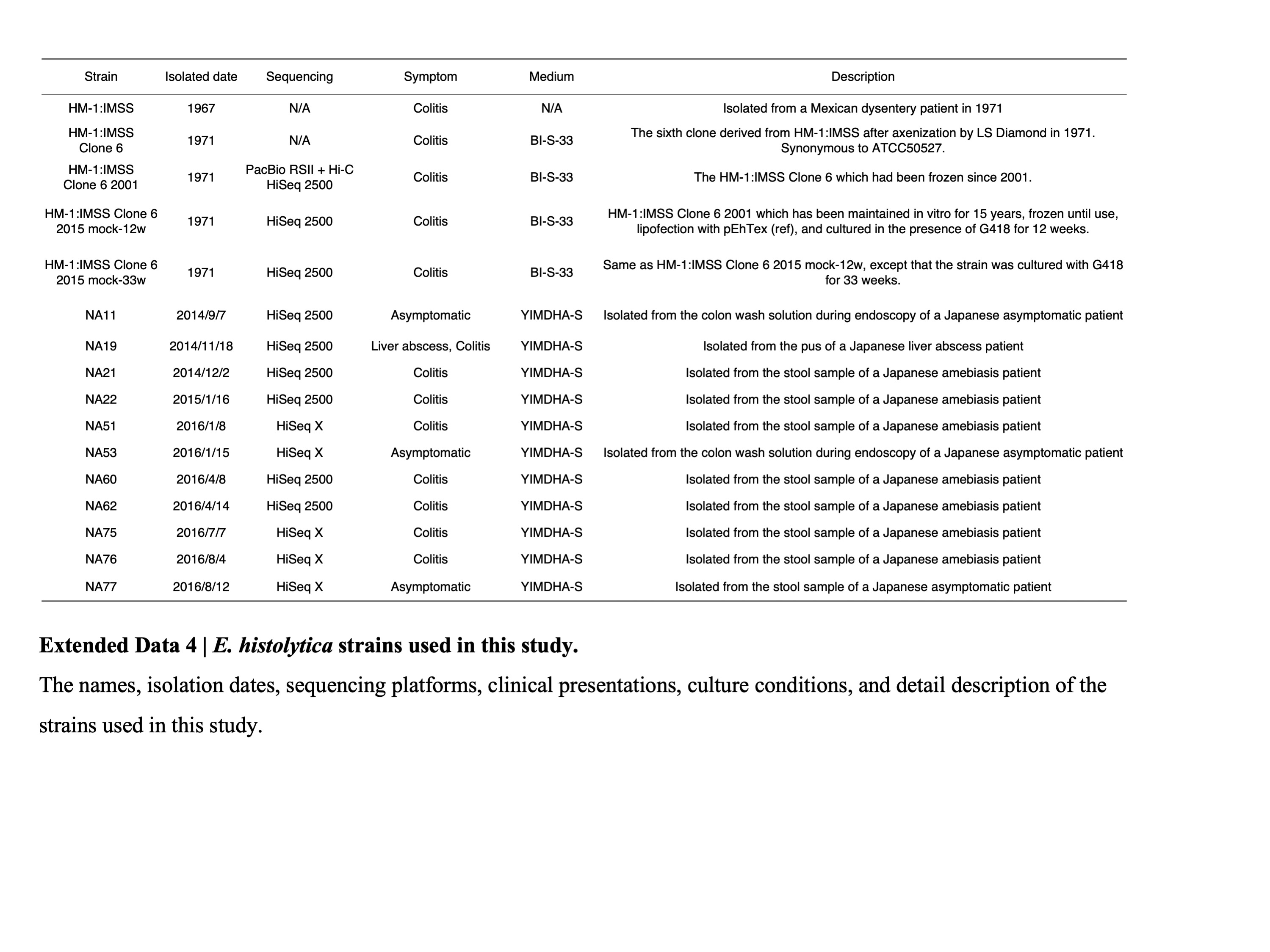

Supplement: Supplementary file 1 — Additional file 1: Extended Data 1-4. [file 12864_2020_7167_MOESM1_ESM.zip › 12864_2020_7167_MOESM1_ESM/extdata4.jpeg]
